# Measuring infectious disease stigma in the German population: Psychometric properties of stigma scales related to COVID-19, Long Covid and non-vaccination in a representative population sample

**DOI:** 10.1186/s13690-026-02100-0

**Published:** 2026-10-30

**Authors:** Jule Menzinger, Carlotta Alpers, Thomas McLaren, Dafina Danqa, Marlene Muehlmann, Silke Schmidt, Samuel Tomczyk

**Affiliations:** 1 Institute of Psychology, Department Health and Prevention, University of Greifswald, Greifswald, Germany; 2 Partner site Greifswald/Rostock, German Centre for Child and Adolescent Health (DZKJ), Greifswald, Germany

**Keywords:** COVID-19, Long Covid, Vaccination, Internalized Stigma, Public Stigma, Scale validation, Psychometric Properties, Germany

## Abstract

**Background:**

The COVID-19 pandemic was widely accompanied by stigma towards certain groups of people, which can diminish efforts of disease control and exacerbate negative impacts of the disease itself. Research on COVID-19-related stigma in the German population is scarce and, internationally, is lacking validated and standardized measurement instruments. To address this issue, this study tested the psychometric properties of a set of scales to measure COVID-19-related stigma in a representative sample of the German population.

**Methods:**

Overall, 1960 people (M(SD)age = 49.8(17.3) years, 55.8% female) participated in a nationally representative cross-sectional survey in Germany. Six scales were tested, assessing public attitudes towards people who have had COVID-19 (COVID-PSS), those who had the disease multiple times (MI-PSS), people with Long Covid (LC-PSS) and people who chose not to get vaccinated (nonVac-PSS). We further assessed internalized stigma of individuals, who have had COVID-19 at least once (COINS) and those who chose not to get vaccinated (nonVac-IS). All scales were tested for their intended factorial structure through confirmatory factor analysis, convergent and discriminant validity and reliability.

**Results:**

All scales, excepting the scale for internalized stigma of unvaccinated individuals, showed good to excellent fit indices and factor loadings, supporting their intended factorial structure. Convergent and discriminant validity were established for the public stigma scales. The COINS did not converge with a measure of quality of life, but with depressive symptoms, whereas the nonVac-IS did not show convergence with presumed related constructs. Reliability coefficients showed good internal consistency among the items.

**Conclusion:**

Five out of the six scales proved to be valid tools to measure stigmatizing attitudes related to COVID-19, Long Covid and non-vaccination in a German sample. The scales hold promises for monitoring stigma during infectious disease outbreaks and informing the development of public health interventions to counteract health-related stigma.

**Supplementary Information:**

The online version contains supplementary material available at https://doi.org/10.1186/s13690-026-02100-0.

**Table:** 

Text box 1. Contributions to the literature
• This study provides the first validated, standardized measurement instruments for COVID-19-related stigma in a representative German population sample (*n* = 1960). • It addresses a critical gap by offering psychometrically sound scales to measure public stigma towards COVID-19 infection, Long Covid, and non-vaccination, as well as internalized stigma among affected individuals.
• The validated instruments enable systematic monitoring of pandemic-related stigma, which can reduce intention to test and seek treatment, thereby undermining disease control efforts. • The findings support the use of these brief, reliable scales for public health surveillance during infectious disease outbreaks and for evaluating anti-stigma interventions in diverse populations.

## Background

Stigmatization, as a process of labelling, negatively stereotyping and separating (groups of) people based on certain characteristics, has been shown to occur in the context of infectious diseases such as HIV/AIDS, Ebola, Tuberculosis, Zika, SARS and others [1, 2]. Such health-related stigma was also prevalent during the recent COVID-19 pandemic, where stigmatizing attitudes occurred towards those infected with the virus or associated with the disease [3–6]. According to Yuan et al. [2], enacted and perceived stigma related to COVID-19 had an overall prevalence of 35%, estimated over 29 studies that were conducted worldwide between 2020 and 2021, in samples of community populations, healthcare workers and patients with COVID-19. Ransing et al. [7] applied the Health Stigma and Discrimination Framework (HSDF) [8] to the context of the COVID-19 pandemic, outlining manifestations of stigma, its development and consequences. They describe COVID-19-related stigma manifestations in the form of experiences (e.g., internalized, perceived, experienced or associative stigma) and practices such as discriminatory attitudes, social rejection or prejudice towards diseased people as well as structural factors (e.g., difference in income, housing, education) that can exacerbate or diminish stigma and its effects.

COVID-19-related stigma has been shown to affect certain groups of people. For instance, individuals who have had COVID-19 faced discrimination or social exclusion due to missing information and irrational fears of contagion [9, 10]. Stigmatizing attitudes towards the diseased might be altered when confronted with people who repeatedly contracted COVID-19, potentially being perceived as careless or irresponsible. To date, there has been no research on this potential mechanism. Persons with Long Covid faced further challenges, such as scepticism about the validity of their symptoms or their ability to contribute to work and society [11]. Additionally, unvaccinated individuals have often been labelled as irresponsible or a threat to public health, fuelling prejudice and discrimination towards this group during the pandemic [12]. These nuanced experiences highlight the importance to consider specific subgroups when studying and addressing stigma related to COVID-19 or infectious diseases in general. The consequences of such COVID-19-related stigma are far-reaching. According to Fischer et al. [1], it can have a negative impact on an individual (e.g., internalized stigma can lead to social isolation, negative mental health) or organizational level (e.g., reduced productivity), and obstruct efforts to prevent disease transmission (e.g., delay or prohibit self-testing, diagnosis and treatment-seeking).

To counteract stigma, it is paramount to swiftly and accurately recognize and monitor it during disease outbreaks [13, 14] to allow for tailored and timely public health action. Internationally, extensive research has been conducted on COVID-19-related stigma throughout the pandemic. A major limitation to these studies was the common use of unvalidated stigma scales or single-item measures to assess stigma [2]. For the German population, few studies have examined experiences or practices of stigma during the pandemic, focusing primarily on subgroups, such as healthcare workers [6], people of Asian descent [15], or COVID-19 survivors [9, 10]. One study focused on the form of internalized stigma related to COVID-19 [16]. As part of the study of Bor et al. [12], participants from Germany showed higher negative attitudes towards individuals of their respective outgroup regarding vaccination status during the COVID-19 pandemic.

The limited research on COVID-19-related stigma in the German population might be due to a lack of psychometrically sound instruments for measurement. Consequently, public health officials and health research are in need of validated tools that allow for a long-term monitoring of COVID-19-related stigma (including acute disease symptoms, Long Covid, and people without a vaccination) and can serve as a blueprint for future infectious disease outbreaks. Our study aims to address this by developing and validating a set of scales to assess COVID-19-related public stigma toward four target groups: (1) individuals who have had COVID-19 once, (2) individuals who have had COVID-19 repeatedly, (3) individuals diagnosed with Long COVID, and (4) unvaccinated individuals. In accordance with the HSDF [7, 8], we will also test two instruments for measuring the experience of internalized stigma in (1) individuals who have had COVID-19 at least once and (2) individuals who chose not to be vaccinated against the virus, as both groups faced high levels of stigmatizing attitudes throughout the pandemic, potentially leading to an internalization of such attitudes.

## Method

### Study design and sampling

A cross-sectional, population-based online survey was carried out between October and December 2023 in Germany. Adults above the age of 18 years were recruited through the Talk Online Panel, based on quota sampling, reflecting the distributions of gender, age and region of residence in the German population. Participants entered the survey platform SoSci Survey [17] via link, where they received information about the study aims and the anonymous processing of their data. Only after giving informed consent to participate in the study, participants were able to begin the survey.

### Questionnaires

The survey was administered in German and included several socio-demographic variables (e.g., age, gender, region, education, profession) and questionnaires assessing participants’ mental health (PHQ-9 [18]; GAD-7 [19]), health-related quality of life (EUROHIS-QOL; [20]), stigmatizing beliefs, and experiences with COVID-19. Based on a systematic literature review [21], six scales measuring public and internalized stigma related to COVID-19 were selected, translated and adapted for use in the German population.

### Translation and adaptation procedure

Three instruments were originally developed for the COVID-19 pandemic [12, 22, 23] and, therefore, mainly required translation. The scale from Nochaiwong et al. [23] was used in its original and an adapted form, to assess attitudes toward individuals with singular and repeated COVID-19 infections, respectively. Two further scales measuring mental illness-related stigma were adapted by replacing terms referring to mental illness with the terms “Long Covid” [24] or “unvaccinated” [25]. All measures were translated from their English version into German if they were not already available in German. Translations were conducted by three project members, one of whom was a bilingual native speaker (English/German) and two of whom were native German speakers fluent in English. The complete questionnaire underwent cognitive debriefing through interviews (*n* = 13) and an online pretest (*n* = 241), resulting in final adjustments. These included adapting the instructions to the post-pandemic context, aligning the response options of the Likert scales and, for the *COVID-19 Public Stigma Scale* (COVID-PSS) [23], splitting one item into two, depicting the separate aspects of individuals being a burden to society and to family. Adaptations and translation of the scales can be found in the supplementary material (Tables A-F).

### Public stigma scales

Public stigma was operationalized as the agreement of participants with stereotypes towards people with COVID-19-related characteristics. Stigma towards people infected with COVID-19 was measured using the COVID-PSS developed and tested by Nochaiwong et al. [23]. The original scale includes ten items and three factors: stereotype (three items), prejudice (three items) and fear (four items). Items contained statements about persons with COVID-19, and participants were asked to rate their agreement on a 5-point Likert scale ranging from 1 “don’t agree at all” to 5 “agree completely”, with higher scores indicating higher stigmatizing attitudes. This Likert scale was used for all public stigma measures. After cognitive debriefing, one item from the prejudice factor (Item 5: “Most people infected with COVID-19 are a burden to their families and society.”) was divided into two items to separately assess the burden to families and society. Face and content validity of the scale were established in the original study, and internal consistency ranged between coefficient alpha = 0.76 and 0.85 for the subscales and total scale.

To measure public stigma towards people who repeatedly fell ill with COVID-19 (MI-PSS), we adapted the COVID-PSS by replacing the respective terms for the target group with: “people who repeatedly fall ill with COVID”. Items from the fear factor were excluded since they specifically referred to a person currently ill with COVID-19, except for item 7 (“Most people who repeatedly fall ill with COVID are a danger to other people”), which was adaptable to the new target. Two new items were formulated, assessing a behavioral component of stigma, resulting in an assumed three-factor structure: stereotype (three items), prejudice (four items), and behavior (two items).

Public stigma towards Long Covid patients (LC-PSS) was assessed with four adapted items from Schomerus et al. [24], originally measuring public stigma towards mental health patients. As disbelief in the illness has been mentioned as stigmatizing by Long Covid patients [11], we further added a fifth item, stating: “Long Covid is not a real illness”. The original items were conceptualized to load on one factor, and the scale was therefore expected to fit a single factor model.

Lastly, we included a scale with four items from Bor et al. [12] to measure exclusionary attitudes towards unvaccinated individuals (nonVac-PSS). The original yes/no scale was adapted to the 5-point Likert scale for consistency among the public stigma scales’ ratings. We also expect the items to load on a single factor.

### Internalized stigma scales

Internalized stigma was assessed through the *Covid-19 internalized stigma scale* (COINS) [22], containing 13 items and three factors: self-perception (seven items), close relations (three items) and social life (three items). The items contained statements about self-stigma due to having had COVID-19, and participants ranked their agreement on a 4-point Likert scale ranging from 1 “not at all” to 4 “a lot”. Construct validity of the scale was established in the original study and showed overall good internal consistency with coefficient alpha = 0.84 to 0.92 for the subscales and total scale. Internalized stigma of people who are not vaccinated against COVID-19 (nonVac-IS) was measured with six items adapted from the *Internalized Stigma of Mental Illness Scale* (ISMI-9) [25]. We further included two self-formulated items that were guided by items from the long version of the ISMI [26]. The nonVac-IS reflects on social withdrawal, feelings of alienation and shame due to being unvaccinated. Agreement with the statements was ranked on a 4-point Likert scale ranging from 1 “strongly reject” to 4 “strongly agree”, with higher scores indicating higher internalized stigma. The items will be tested for a single-factor model.

During cognitive debriefing, some participants experienced difficulties in rating their agreement to certain statements on the COINS, MI-PSS, and nonVac-PSS. To minimize drop-out in the final study due to difficulties, an option to answer “not applicable” was incorporated in the final questionnaire, based on the original COINS [22]. The option was defined as a missing value, as participants actively opted against other options, and we decided to not artificially assign a value by imputing missing data points. All cases with missing values due to choosing “not applicable” or not answering an item were excluded from analyses of validity and reliability for the respective scales. Descriptive statistics of all items are available in the supplementary material (Tables G and H).

### Internal structure validity

All scales were tested for their intended factorial structure using confirmatory factorial analysis (CFA), which was conducted in R [27] using the lavaan package developed by Rosseel [28]. To establish the metric for the analysis, we set the factor variance to 1. Fit indices were calculated to assess model fit based on Maximum Likelihood (ML) estimation with Satorra-Bentler correction [29] if multivariate normality was not given. Goodness-of-fit measures included the comparative fit index (CFI) and Tucker Lewis Index (TLI) indicating the proportionate improvement in fit, compared to a null-model [30], root mean square error of approximation (RMSEA) and Standardized Root Mean Square Residual (SRMR), evaluating how well the theoretical model reproduces the sample data [30]. A good model fit is indicated by CFI and TLI values close to 0.95, RMSEA values close to 0.06, and SRMR values close to 0.08, thereby minimizing Type II error rates [30]. Chi-Square will not be further evaluated due to its sensitivity to large sample sizes [31]. Standardized factor loadings should be 0.50 or higher. In cases where a model did not show adequate fit based on fit indices and factor loadings, we took modification indices into account and tested models with theoretically plausible respecifications [32].

### Convergent and discriminant validity

Convergent and discriminant validity were evaluated through Spearman’s correlation coefficient (r_s_), to test for associations between the stigma scales and related or unrelated constructs, respectively. We oriented our interpretation of r_s_ on Leclezio et al. [33] and expect to find at least moderate relationships with r_s_ >0.30 among related constructs and no or negligible relationship of r_s_ <0.20 for theoretically unrelated constructs. The same thresholds account for expected negative correlations. Convergent validity of the internalized stigma scales was assessed through correlations of the nonVac-IS and COINS with a measure of quality of life (EUROHIS-QOL) [20] and depressive symptoms (Patient Health Questionnaire-9 [PHQ-9]) [18]. For the PHQ-9 we expect a weak to moderate positive correlation [34], whereas for the EUROHIS-QOL, a negative correlation would be indicative for convergent validity, as higher internalized stigma shows to be associated with a lowered quality of life [35, 36]. To establish discriminant validity, we estimated correlations of the internalized stigma scales with the *Social Distance Scale* (SDS) [37], as there should be no substantial associations between internalized stigma related to COVID-19 and the desire for social distance towards people with mental illness. The public stigma scales were correlated with each other to assess convergent validity, as they all aim to measure the construct of public stigma, but with different target populations. Among the COVID-PSS and MI-PSS higher correlations are expected due to the overlap of items and their target group of people with COVID-19 infections. For the LC-PSS and nonVac-PSS, we hypothesize somewhat weaker but significant positive correlations because the target populations are more different from one another. Discriminant validity was evaluated through correlations of the public stigma scales with the EUROHIS-QOL and the PHQ-9, assuming no associations between public stigma and depressive symptoms or quality of life.

### Questionnaire reliability

Reliability of the item sets was assessed by calculating McDonald’s omega ω [38] as a measure of internal consistency among items. Based on the assumption that our scales are congeneric, estimation of the commonly used coefficient alpha would be inaccurate due to its assumption of tau equivalence of the scales [39]. McDonald’s ω was calculated for each factor using the MBESS package [40]. Acceptable reliability of the scales would be indicated by ω > 0.70, good internal consistency is indicated by ω > 0.80 [41].

## Results

### Sample characteristics

A total of *N* = 2972 persons completed the online survey. We excluded *n* = 541 cases that did not pass a quality check item and *n* = 422 speeders falling below our minimum response time of 11.44 min needed to complete our survey, based on the formula provided by Andreadis [42]. Finally, *n* = 49 cases were excluded, which we identified as having been completed by bots, based on written answers. This resulted in a final total sample of *N* = 1960 participants. The mean age was 49.8 years (SD = 17.3), with 55.8% (1093/1960) female participants. More than half of the respondents had COVID-19 at least once (55.7%; 1094/1960), of which *n* = 163 have a diagnosis of Long Covid and *n* = 260 indicated having had COVID-19 multiple times since the beginning of the pandemic. Most participants (85.4%; 1674/1960) reported having had at least one vaccination against the virus. Prior to statistical analyses we evaluated missing data based on true missing values (i.e., when participants did not answer an item at all). Overall, there were < 1% (16/1960) cases with missing data on single items of the relevant scales (five items of the EUROHIS-QOL, two items of the COVID-PSS, one item of the MI-PSS). Due to the small number, a missing analysis was not necessary [43], and cases were deleted listwise for analyses including the respective scale.

### Confirmatory factor analysis

**Fig. 1:**
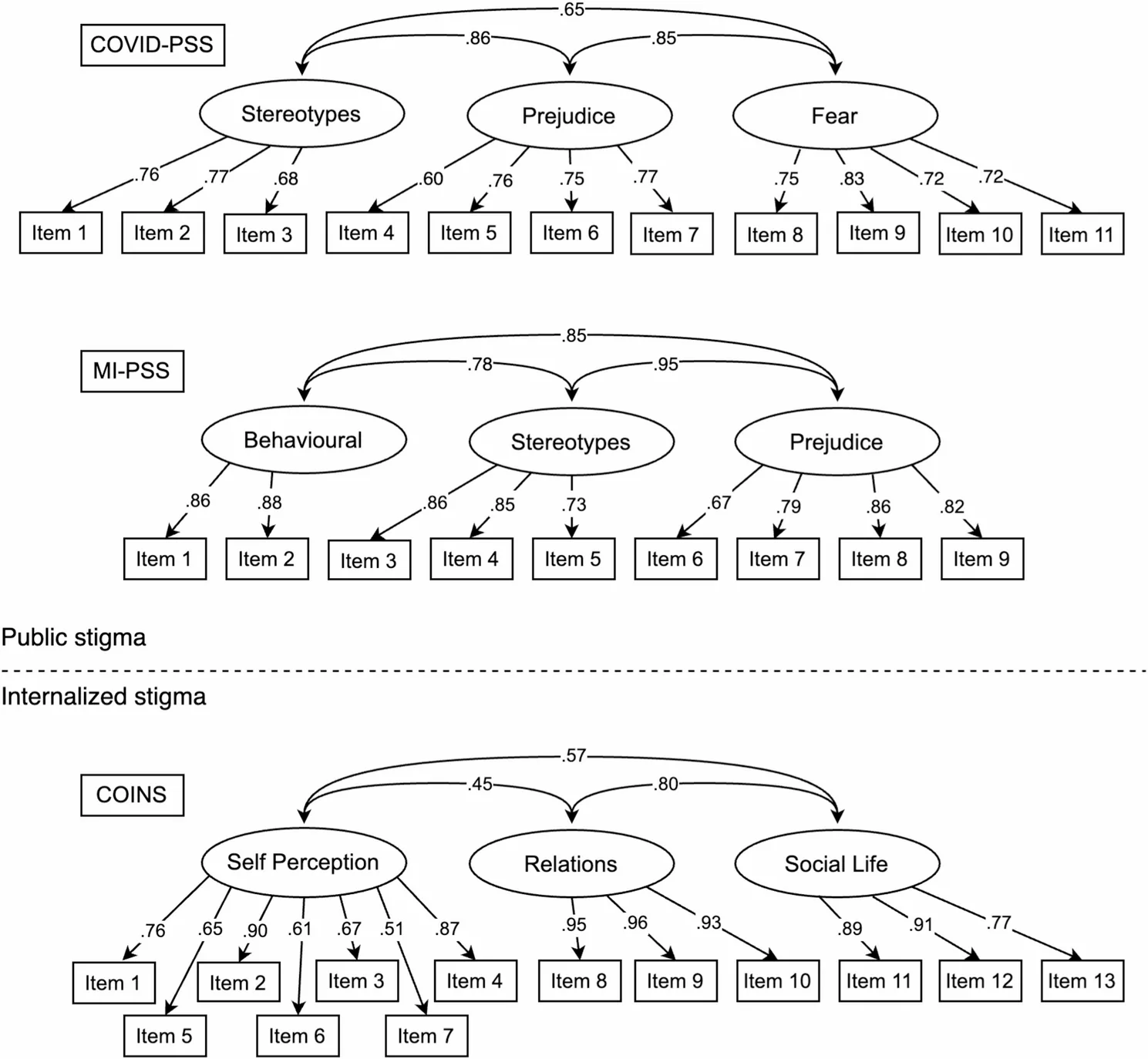
Factor models with standardized factor loadings and factor covariances for the three-factorial stigma scales. Note. Factor-loadings indicated through single-headed arrows; covariances indicated through double-headed arrows; COVID-PSS = COVID-19 Public Stigma Scale; MI-PSS = Multiple Infections Public Stigma Scale: COINS = COVID-19 Internalized Stigma Scale

Results of the maximum likelihood confirmatory factor analysis (see Tables 1, 2 and 3) revealed an overall good fit of the hypothesized three-factor and single-factor models of the public stigma scales. Fit indices ranged between CFI = 0.95–0.99, TLI = 0.93–0.99, RMSEA = 0.04–0.08 and SRMR = 0.007–0.05. All items further exceeded standardized factor loadings of 0.50, showing good to excellent loadings on their respective factor [44]. For the MI-PSS and COVID-PSS, correlation between the stereotypes and prejudice factor was 0.95 and 0.85, respectively (Fig. 1), suggesting a high overlap of the factors. Analysis of the three-factor COINS model also showed a good model fit and good to excellent factor loadings. The single-factor model of the nonVac-IS resulted in an unsatisfying model fit for the single-factor model and factor loadings ranged widely from poor to excellent (0.41–0.74).

**Table 1:** Robust maximum-likelihood goodness-of-fit indices for the scales measuring COVID-19 related public and internalized stigma

Scale	*n*	² (df)	CFI	TLI	RMSEA	SRMR
Public Stigma Scales						
COVID-PSS	1958	572.91 (41)	0.95	0.93	0.08	0.05
MI-PSS	1568	213.5 (24)	0.98	0.97	0.07	0.02
LC-PSS	1960	61.66 (5)	0.99	0.98	0.07	0.02
nonVac-PSS	1670	10.53 (2)	0.99	0.99	0.04	0.007
*Internalized Stigma Scales*						
COINS	824	470.73 (62)	0.96	0.95	0.08	0.08
nonVac-IS	266	79.09 (20)	0.90	0.86	0.10	0.06

*CFI* Comparative fit index, *TLI* Tucker-Lewis index, *RMSEA* Randomized standard error approximation, *SRMR* Standardized root-mean-square residual, *COVID-PSS* COVID-19 Public Stigma Scale, *MI-PSS* Multiple Infections Public Stigma Scale, *LC-PSS* Long COVID Public Stigma Scale, *nonVac-PSS* Unvaccinated Public Stigma Scale, *COINS* COVID-19 Internalized Stigma Scale, *nonVac-IS* Unvaccinated Internalized Stigma Scale

**Table 2:** Means, standard deviations, standardized factor loadings and internal consistency of the public stigma scales

Scale	M	SD	factor loadings			McDonald‘s ω [95%-CI]^a^
			1	2	3	
COVID-PSS						
*Most people infected with COVID…*						
1. do not take care of their health.	2.09	1.04	0.76			stereotype 0.78 [0.76, 0.80]
2. do not follow expert medical advice.	2.43	1.08	0.77			
3. like to party or socialize often.	2.37	1.11	0.68			
4. are contaminated with germs.	2.41	1.19		0.60		prejudice 0.81 [0.79, 0.82]
5. are a burden to their families.	2.26	1.10		0.76		
6. are a burden to society.	2.10	1.09		0.75		
7. are socially irresponsible.	2.08	1.09		0.77		
8. are a danger to other people.	2.69	1.21			0.75	fear 0.84 [0.83, 0.86]
9. I fear people infected with COVID.	2.37	1.22			0.83	
10. I fear people who are at risk of COVID infection even if they have not been infected yet.	2.09	1.11			0.72	
11. I fear being infected with COVID if I live in a community with people who are infected with COVID.	2.93	1.33			0.72	
MI-PSS			1	2	3	
*Most people who repeatedly fall ill with COVID…*						
1. should be condemned more than people who only fall ill with COVID once.	1.51	0.94	0.86			behaviour 0.86 [0.83, 0.88]
2. should be ostracized/shunned more than people who have only had COVID once.	1.58	0.98	0.88			
3. do not take care of their health.	2.04	1.18		0.86		stereotype 0.86 [0.83, 0.88]
4. do not follow expert medical advice.	2.18	1.19		0.85		
5. like to party or socialize often.	2.34	1.23		0.73		
6. … are contaminated with germs.	2.07	1.23			0.67	prejudice 0.86 [0.84, 0.87]
7. … are a burden to their families and society.	2.19	1.20			0.79	
8. are socially irresponsible.	1.90	1.14			0.86	
9. are a danger to other people.	2.27	1.25			0.82	
LC-PSS				**1**		
1. Persons with Long Covid are to blame for their problems.	1.49	0.88		0.80		0.86 [0.84, 0.88]
2. Long Covid usually result from a weak character.	1.36	0.78		0.84		
3. Persons with Long Covid only have to pull themselves together in order to get well.	1.64	1.01		0.75		
4. One of the main causes of Long Covid is lack of self-discipline.	1.50	0.91		0.86		
5. Long Covid is no real illness.	1.66	1.09		0.53		
nonVac-PSS				1		
1. I would be unhappy if someone who is not vaccinated against COVID married one of my close relatives.	1.91	1.22		0.82		0.89 [0.89, 0.91]
2. I would be afraid that someone who is not vaccinated against COVID infected me or my family with COVID.	2.45	1.40		0.78		
3. I think someone who is not vaccinated against COVID is unintelligent.	2.24	1.34		0.84		
4. I think someone who is not vaccinated against COVID is untrustworthy.	2.07	1.23		0.90		

*COVID-PSS* Covid-19 Public Stigma Scale, *MI-PSS* Multiple Infections Public Stigma Scale, *LC-PSS* Long Covid Public Stigma Scale, *nonVac-PSS* Unvaccinated Public Stigma Scale; All items were answered on a five-point Likert scale from *do not agree at all* (1) to *agree completely* (5)

^a^ 95% confidence interval based on 5000 bootstrap iterations

**Table 3:** Means, standard deviations, standardized factor loadings and internal consistency of the internalized stigma scales

Scales	M	SD	factor loadings			McDonald‘s ω [95%-CI]^a^
			1	2	3	
COINS						
1. How much do you feel you are not as good a person as others because you have had Covid-19?	1.35	0.79	0.76			self perception 0.88 [0.86, 0.90]
2. How much do you feel ashamed that you have had Covid-19?	1.21	0.63	0.90			
3. How much do you feel that it is your fault that you have had Covid-19?	1.32	0.71	0.67			
4. How much do you feel embarrassed that you have had Covid-19?	1.21	0.62	0.87			
5. How much have you avoided telling others that you have had Covid-19?	1.34	0.75	0.65			
6. How much have you stopped socializing with people because of their negative reactions to you having Covid-19?	1.48	0.93	0.61			
7. How much do you feel uncomfortable to go anywhere outside of your house because you have had Covid-19?	1.64	1.00	0.51			
8. How much would you understand your family members if they avoided you because you have had Covid-19?	1.89	1.18		0.95		close relations 0.95 [0.94, 0.96]
9. How much would you understand friends if they avoided you because you have had Covid-19?	1.88	1.17		0.96		
10. How much would you understand your neighbors if they avoided you because you have had Covid-19?	1.88	1.16		0.93		
11. How much would you agree with employers if they would not employ you because you have had Covid-19?	1.50	0.95			0.89	social life 0.89 [0.87, 0.91]
12. How much would you understand people if they excluded you from areas of public life (e.g., sporting centers, clubs, cultural events, voluntary groups) because you have had Covid-19?	1.58	1.01			0.91	
13. How much would you understand your doctor if he/she was not available to see you because you have had Covid-19?	1.49	0.94			0.77	

nonVac-IS	M	SD		1		ω [95%-CI]
1. Clichés about unvaccinated people apply to me.	1.45	0.79		0.53		0.77 [n.a.]^b^
2. Negative stereotypes about unvaccinated people isolate me from others.	1.69	0.93		0.59		
3. I sometimes feel out of place because I am unvaccinated.	1.34	0.70		0.74		
4. Being around people who are vaccinated makes me feel like I don’t belong.	1.54	0.84		0.68		
5. Vaccinated people can’t possibly understand me.	2.30	1.00		0.41		
6. No one would be interested in getting close to me because I am unvaccinated.	1.45	0.78		0.61		
7. In some social situations, I am embarrassed to be unvaccinated.	1.25	0.59		0.64		
8. Being unvaccinated has had a negative impact on my life.	1.56	0.88		0.49		

*COINS* Covid-19 internalized stigma scale, *nonVac-IS* Unvaccinated Internalized Stigma Scale. all items were answered on a four-point Likert scale from *not at all* (1) to *a lot* (4) for the COINS and from *strongly reject* (1) to *strongly agree* (4) for the nonVac-IS

^a^95% confidence interval based on 5000 bootstrap iterations

^b^Confidence interval could not be calculated with modelled error covariances

Modification indices proposed allowing for error covariance between certain items to improve local fit of the unidimensional model. Beginning with the highest modification index of 13.09, we tested a model allowing for error covariance between items 1 (“Clichés about the unvaccinated apply to me”) and 6 (“No one would be interested in getting close to me because I am unvaccinated”). An overlap of the items might occur, as both reflect self-blame and acceptance of societal stereotypes about being unvaccinated, pointing to a shared underlying emotional experience. The model resulted in a better, yet unsatisfactory fit (CFI = 0.92, TLI = 0.89, RMSEA = 0.09, SRMR = 0.06). For this model, a modification index of 10.71 was given, suggesting to further model error covariance between item 2 (“Negative stereotypes about unvaccinated people isolate me from others”) and item 5 (“Vaccinated people can’t possibly understand me”). Both items share an underlying sentiment of alienation, where ‘the others’ (vaccinated people) are the ones to blame for this circumstance. This model resulted in an acceptable model fit, with CFI = 0.94, TLI = 0.91, RMSEA = 0.08, and SRMR = 0.05, but factor loadings still ranged widely between 0.36 to 0.77, with two falling below our cut-off of 0.50. An exploratory parallel analysis and scree plot did not reveal another plausible factor solution.

### Convergent and discriminant validity

To assess construct validity, correlations of the public and internalized stigma scales are reported in Table 4. All correlations between the public stigma scales were highly significant, with the highest correlation between the COVID-PSS and MI-PSS. The LC-PSS showed a strong correlation with the MI-PSS, but weak or negligible correlations with the COVID-PSS and nonVac-PSS. Correlations between the public stigma scales and the EUROHIS-QOL or PHQ-9 were close to zero, reflecting discriminant validity. The COINS had a significant but weak relationship with the PHQ-9 (*r* = .27, *p* < .001), and no correlation with the EUROHIS-QOL (*r* = − .01, *p* = .85) was present. For the nonVac-IS correlations with the PHQ-9 (*r* = .14, *p* = .02) and EUROHIS-QOL (*r* = − .11, *p* = .07) were both negligible. Concerning discriminant validity, a significant but negligible negative relationship was present with the SDS (*r*(COINS) = 0.018 and *r*(nonVac-IS) = 0.12, *p* < .001).

**Table 4:** Spearman’s correlation coefficient matrix for the public stigma scales

Scale	EUROHIS-QOL	PHQ-9	COVID-PSS	MI-PSS	LC-PSS
COVID-PSS	-0.08***	0.12***	1		
MI-PSS	0.01	0.08**	0.7***	1	
LC-PSS	0.06**	0.03	0.25***	0.42***	1
nonVac-PSS	0.05	0.06*	0.52***	0.53***	0.12***

*COVID-PSS* COVID-19 Public Stigma Scale, *MI-PSS* Multiple Infections Public Stigma Scale, *LC-PSS* Long Covid Stigma Scale, *nonVac-PSS* Unvaccinated Public Stigma Scale, *EUROHIS-QOL* European Health Interview Survey - Quality of Life Index, 8 Item Index, *PHQ-9* Patient Health Questionnaire-9

*p < .05, **p < .01, ***p < .001

### Reliability

Except for the COVID-PSS stereotype factor with an acceptable coefficient of ω = 0.78 and the nonVac-IS with ω = 0.77, all scales and subscales obtained McDonald’s ω of 0.80 and above, indicating overall good to excellent consistency and reliability of the scales (see Tables 2 and 3) [41].

## Discussion

Infectious disease stigma is a key concern for public health, as it may impede efforts to contain disease outbreaks, due to associations with behavioral health, like adherence to safety regulations [45]. It can also negatively affect psychological and social health of those being stigmatized [1]. Stigma monitoring therefore should be an integral part of pandemic response strategies, for which well-validated instruments are essential [13]. This study aimed to aid this goal by developing and psychometrically testing a set of scales to measure internalized and public stigma related to the COVID-19 pandemic, among a sample of German adults.

For all public stigma scales, internal structure validity could be established through confirmatory factor analysis, resulting in overall good to excellent fit-indices and factor loadings for the expected three-factorial structure of the COVID-PSS and MI-PSS as well as the single-factor model of the LC-PSS and nonVac-PSS. The CFA further revealed high correlations between the two factors *stereotype* and *prejudice* on the COVID-PSS and MI-PSS, suggesting a potential overlap. Results from the original scale’s development showed notably lower correlations between the two factors [23]. We attribute this to the conceptual relationship between stereotypes and prejudices with stereotypes being conceptualized as the cognitive part of group categorization processes, preceding or justifying the formation of prejudice [46]. Paterson et al. [47] further model prejudice as an endorsement of stereotypes, linking them to negative thoughts and feelings. Since the original scale has been developed and tested in an early stage of the COVID-19 pandemic, stereotypes towards people with COVID-19 might have been more salient than prejudices at that time, which is supported by lower mean and median values for the prejudice compared to the stereotype items as well as a lower correlation between the factors [23]. With our study having been conducted three years after the onset of the pandemic, the high correlation between the factors *stereotype* and *prejudice* might reflect on the dynamic of stereotypes developing into prejudices over the course of time. If a person persistently holds stereotypes towards the group of people infected (repeatedly) with COVID-19, it is likely that these have developed into prejudices over the course of the pandemic. Likewise, a person that didn’t hold or revised their own stereotypes towards those infected, will not have developed prejudices. In both cases, this would result in similar levels of stigma on both factors over time and therefore a higher correlation. Maintaining the original structure of the scale could be useful in monitoring the development and interplay of stereotypes and prejudice over the course of an infectious disease outbreak. Therefore, we favor retaining the three-factorial structure, especially when applying the scale in early stages of an outbreak. Further examination of the scale could include analyses on the influence of time of measurement and other possible confounding influences explaining the close relation of the factors.

Convergent and discriminant validity were established through positive moderate to high correlations among the public stigma scales, except for the LC-PSS, which had a negligible association with the nonVac-PSS. While the target groups of the LC-PSS, MI-PSS, and COVID-PSS all share the experience of having been infected with COVID-19 at some point, similar drivers and facilitators likely contribute to the stigma they face. However, unlike developing Long Covid, the decision not to be vaccinated is (mostly) voluntary, an important distinction that may lead to different manifestations of stigma. Vaccination, which can be seen as “a social contract wherein getting vaccinated is the morally right behavior” [48], is related to social norms and moral judgement as drivers of stigma. One study shows that during the COVID-19 pandemic, unvaccinated persons were perceived by the public as lacking civility and not caring enough about their relatives’ health [49]. In the case of Long Covid there is only limited evidence on potential drivers of stigmatizing attitudes, but initial results indicate that limited knowledge or misconceptions about the disease are leading causes for stigma towards Long Covid patients [11, 50]. These contrasting drivers – social condemnation vs. misconceptions – could explain the difference in the prevalence of stigma towards the two target groups, resulting in low associations.

Depressive symptoms and quality of life had no or negligible relationship with the public stigma scales, demonstrating discriminant validity. Internal consistency on the factorial level was high in our sample. The results indicate that the four public stigma scales are overall suitable to measure stigmatizing attitudes of the public towards the four different target populations.

Concerning the scales measuring internalized stigma related to COVID-19, confirmatory factor analysis supported the expected three-factorial structure of the COINS. Confirmatory factor analysis of the nonVac-IS resulted in an acceptable fit, with partly unsatisfying factor loadings, after modelling for error covariances between items 1 and 6 and items 2 and 5. Construct validity could not be fully established through our analysis, as the scales did not converge with the EUROHIS-QOL, assessing quality of life. Our assumption of an association between internalized stigma and a lowered quality of life was based on previous research in populations affected by attention-deficit/hyperactivity disorder (ADHD) [36] or schizophrenia [35]. In the area of mental disorders, the association could be stronger due to the chronic nature of mental illnesses and thus the associated internalization of stigma, exacerbating the negative impact on quality of life. Extrapolating this to internalized stigma in the context of COVID-19 as an acute condition or choosing not to get a vaccination may not be appropriate due to different underlying mechanisms that could explain an association with quality of life. Contrary to the COINS, the nonVac-IS did not converge with the PHQ-9 either, showing a significant but negligible correlation that could be due to the heterogeneous item structure of the nonVac-IS (see below). Reliability assessment of the COINS demonstrated good internal consistency among the items, the reliability of the nonVac-IS was acceptable.

Overall, our results do not support psychometric validation of the nonVac-IS. To our knowledge, no studies on internalized stigma among unvaccinated individuals existed at the time of data collection, and our scale was therefore adapted from the short-form ISMI, a questionnaire developed to measure internalized stigma in the context of mental health [26]. Adapting items is a common practice for developing measurement tools in the context of emerging infectious diseases, yet, this can compromise content validity of the scales, if the items do not adequately reflect on the experiences and beliefs of the population of interest [47]. In case of the nonVac-IS, the items of the ISMI might have been too heterogeneous, to concisely capture central aspects of internalized stigma in individuals who did not get vaccinated, which potentially led to response patterns too inconsistent for a valid factorial structure and measurement tool.

Since vaccines play an important role in pandemic preparedness during infectious disease outbreaks [51], and stigmatizing attitudes towards unvaccinated people were prevalent throughout the COVID-19 pandemic [12, 49], the potential internalization of such attitudes should be monitored and recognized during infectious disease outbreaks. To support this, future research should focus on generating items based on focus groups or interviews with individuals choosing not to get vaccinated, to ensure an adequate representation of their perspectives and lived experiences. Incorporating qualitative data into scale development could further enhance content validity of the measurement tool [52].

### Strengths and limitations

We conducted an online survey and recruited participants through an online panel, ensuring a sample representative of the German population based on the distributions of age, gender, and region. Data from an online panel, however, may result in a slightly biased population, as it limits the population to digitally literate people who have access to the internet and those who have actively registered themselves on the Talk Online Panel. According to the panel, about 100.000 persons from Germany are registered on their website. It can be noted, however, that our sample did include various subgroups especially vulnerable to stigma, such as older or socioeconomically disadvantaged people. A further limitation of our study is the exclusive use of self-reported measures which are susceptible to response bias like social desirability, especially in the field of attitudes [53]. An extension of our research would be to apply a multi-method approach, for example by adding implicit or behavioral measures to the self-report questionnaires, which could also aid in validating the scales. Brief implicit association tests have shown good feasibility in measuring stigmatizing attitudes towards individuals with mental illness [54] and could be adapted to the context of infectious disease stigma in future research. Finally, the development of the scales did not follow a participatory research approach, as we based our instruments on existing stigma scales. Some, such as the nonVac-PS, the COVID-PSS, and the COINS, have been developed and shown to be valid in the context of COVID-19, whereas our scales assessing stigma in the groups of people with Long Covid or unvaccinated individuals were adapted from instruments measuring stigma of mental illness. As discussed above, those items might not sufficiently represent the lived experiences of the stigma surrounding both populations. We would therefore recommend refining the scales or developing new measurement tools, applying a participatory approach to further capture the lived experiences of relevant subgroups with potential risk of stigmatization during infectious disease outbreaks. This would enhance content and external validity [52]. One final limiting aspect that needs to be carefully evaluated in future use of the scales is the incorporation of the option to answer, “not applicable.” As participants actively chose not to select one of the substantive response options, we treated “not applicable” as a missing value rather than imputing a value artificially. This nonetheless resulted in the exclusion of incomplete datasets for some analyses, which may have biased the estimated factor structure and compromised validity. Our main reason for implementing the option in the first place were reports of difficulties in answering some of the items during cognitive debriefing. Therefore, we allowed for an alternative option to minimize overall dropout. Descriptive data shows that some items had higher rates of “not applicable” responses than others, which might reflect on a higher difficulty to respond to the statements. When using the scales in future research, the respective items should be further evaluated qualitatively. Yet, most participants answered all items without using the alternative option or used it for only one or two items per scale. Therefore, it should be carefully examined, if this option needed in future use of the scales or if it should be at least rephrased, to avoid having uninterpretable, artificial missing values in the data.

### Implications

Health-related stigma adversely affects both individual health outcomes and broader societal processes, ultimately undermining disease control efforts. This was evident during the recent COVID-19 pandemic, where stigma was linked to increased testing and vaccination hesitancy [55], diminished healthcare access, and poorer patient outcomes [56]. Ransing et al. [7] conclude that COVID-19-related stigma can lead to reduced psychological well-being, a lowered quality of life, and heightened morbidity and mortality. Despite robust evidence that infectious disease outbreaks are accompanied by stigma, current measurement tools have not kept pace with the rapid evolution of epidemics [47]. To date, no standardized, validated instrument exists that can be universally applied across different diseases and settings [47, 57]. In our study, we adapted and validated existing stigma scales for use in a German sample, demonstrating their transferability across cultural contexts. The findings represent a significant advance toward standardizing stigma measurement in infectious diseases, which, in turn, can enhance the comparability of data across countries. Our scales assess stigma not only related to acute infection but also towards individuals who repeatedly fall ill with a disease, develop a chronic illness after an acute infection or choose not to get vaccinated. The focus on different target groups can be especially helpful in capturing the dynamics of stigmatization over the course of an outbreak. Routine use of these scales to monitor stigma can empower public officials and health institutions to refine outbreak control strategies. By focusing not only on preventing disease transmission but also on reducing stigma, unintended consequences, such as public messaging that creates fear and shame or practices like contact tracing and quarantine that may foster othering [55], can be avoided. The scales also offer a valuable tool for designing and implementing targeted interventions to reduce stigmatizing attitudes and behaviors, thereby supporting disease control and improving social and health outcomes. Future research should continue to explore the applicability of these instruments across different languages, cultural contexts, and infectious diseases.

## Conclusiones

Our study supports the validity, internal consistency, and utility of five out of the six scales we examined for assessing public and internalized stigma related to COVID-19. They hold promise for monitoring stigma related to COVID-19 and for evaluating efforts to prevent and reduce stigma. By examining the manifestations of disease-related stigma and how they evolve over time, steps can be derived that need to be taken to counteract stigma and gain insight into the effectiveness of interventions. In our study, we built upon existing COVID-19 stigma scales and adapted others to fit the COVID-19 context. Their overall sound psychometric properties highlight the potential of easily adaptable measurement instruments to address stigma in emerging public health challenges and become part of pandemic preparedness.

## Supplementary Information

Supplementary Material 1.

## Data Availability

The dataset used in this study will be deposited in a public repository upon acceptance of the manuscript.

## Abbreviations

AIDS: Acquired Immunodeficiency Syndrome
CFA: Confirmatory Factor Analysis
CFI: Comparative Fit Index
COINS: COVID-19 Internalized Stigma Scale
COVID-19: Coronavirus Disease 2019
COVID-PSS: COVID-19 Public Stigma Scale
EUROHIS-QOL: European Health Interview Survey–Quality of Life Index
GAD-7: Generalized Anxiety Disorder–7
HIV: Human Immunodeficiency Virus
HSDF: Health Stigma and Discrimination Framework
ISMI-9: Internalized Stigma of Mental Illness Scale (9-item version)
LC-PSS: Long COVID Public Stigma Scale
MI-PSS: Multiple Infections Public Stigma Scale
ML: Maximum Likelihood
nonVac-IS: Non-Vaccination Internalized Stigma Scale
nonVac-PSS: Non-Vaccination Public Stigma Scale
PHQ-9: Patient Health Questionnaire–9
RMSEA: Root Mean Square Error of Approximation
SARS: Severe Acute Respiratory Syndrome
SDS: Social Distance Scale
SRMR: Standardized Root Mean Square Residual
TLI: Tucker–Lewis Index
