# Supplementary material for: Measuring infectious disease stigma in the German population: Psychometric properties of stigma scales related to COVID-19, Long Covid and non-vaccination in a representative population sample
Source: Arch Public Health. 2026 Oct 30;84:233. doi: 10.1186/s13690-026-02100-0 (PMC13726050; doi:10.1186/s13690-026-02100-0)
Supplement: Supplementary file 1 [file 13690_2026_2100_MOESM1_ESM.docx]

**Supplementary material**

**Measuring infectious disease stigma in the German population: Psychometric properties of stigma scales related to COVID-19, Long Covid and non-vaccination in a representative population sample**

**Table of content**

[**Table A** English and German items of the Covid -19 Public Stigma Scale (COVID-PSS)	2](#_Toc235452844)

[**Table B** English and German items of the Multiple Infections Public Stigma Scale (MI-PSS)	3](#_Toc235452845)

[**Table C** English and German items of the Long Covid Public Stigma Scale (LC-PSS)	4](#_Toc235452846)

[**Table D** English and German items of the Unvaccinated Internalized Stigma Scale (nonVac-PSS)	5](#_Toc235452847)

[**Table E** English and German items of the Covid-19 internalised stigma scale (COINS)	6](#_Toc235452848)

[**Table F** English and German items of the nonVac-IS	7](#_Toc235452849)

[**Table G** Response frequencies and percentages for the response options of the public stigma scales	8](#_Toc235452850)

[**Table H** Response frequencies and percentages of the internalized stigma scales	10](#_Toc235452851)

[**Table I** Goodness-of-fit indices for models with error covariances for the nonVac-IS	11](#_Toc235452852)

[**Table J** Factor loadings of the nonVac-IS with modeled error covariances	11](#_Toc235452853)

[**Table K** Response patterns for the option “not applicable”	12](#_Toc235452854)

# **Table A** English and German items of the Covid -19 Public Stigma Scale (COVID-PSS)

|  | **English** |  | **German** |
| --- | --- | --- | --- |
| 1 | Most people infected with COVID do not take care of their health. | 1 | Die meisten mit COVID infizierten Personen sorgen sich nicht um ihre Gesundheit. |
| 2 | Most people infected with COVID do not follow expert medical advice. | 2 | Die meisten mit COVID infizierten Personen halten sich nicht an ärztlichen Rat. |
| 3 | Most people infected with COVID like to party or socialize often. | 3 | Die meisten mit COVID infizierten Personen feiern gerne und treffen sich oft mit anderen. |
| 4 | Most people infected with COVID are contaminated with germs. | 4 | Die meisten mit COVID infizierten Personen sind mit Keimen kontaminiert. |
| 5 | Most people infected with COVID are a burden to their families.^a^ | 5 | Die meisten mit COVID infizierten Menschen sind eine Belastung für ihre Familien. |
| 6 | Most people infected with COVID are a burden to society.^a^ | 6 | Die meisten mit COVID infizierten Menschen sind eine Belastung für die Gesellschaft. |
| 7 | Most people infected with COVID are socially irresponsible. | 7 | Die meisten mit COVID infizierten Personen sind sozial unverantwortlich. |
| 8 | Most people infected with COVID are a danger to other people. | 8 | Die meisten mit COVID infizierten Personen stellen eine Gefahr für andere Menschen dar. |
| 9 | I fear people infected with COVID. | 9 | Ich habe Angst vor Menschen, die mit COVID infiziert sind. |
| 10 | I fear people who are at risk of COVID infection even if they have not been infected yet. | 10 | Ich habe Angst vor Menschen, die dem Risiko einer COVID-Infektion ausgesetzt sind, auch wenn sie sich noch nicht infiziert haben. |
| 11 | I fear being infected with COVID if I live in a community with people who are infected with COVID. | 11 | Ich habe Angst, mich mit COVID zu infizieren, wenn ich in einer Gemeinschaft mit Menschen lebe, die mit COVID infiziert sind. |

*Note.* Scale ranging from *strongly disagree* (1) to *strongly agree* (5); adaptations made to the original items are underlined.

^a^ The original scale’s item: “Most people infected with COVID are a burden to their families and society” was split into items 5 and 6, to assess the aspects separately.

# **Table B** English and German items of the Multiple Infections Public Stigma Scale (MI-PSS)

|  | **English** |  | **German** |
| --- | --- | --- | --- |
| 1 | Most people who repeatedly fall ill with COVID should be condemned more than people who only fall ill with COVID once.^a^ | 1 | Die meisten Menschen, die öfter an COVID erkranken, sollten stärker verurteilt werden, als Menschen, die nur einmal an COVID erkranken. |
| 2 | Most people who repeatedly fall ill with COVID should be ostracized/shunned more than people who have only had COVID once.^a^ | 2 | Die meisten Menschen, die öfter an COVID erkranken, sollten stärker ausgegrenzt/gemieden werden als Menschen, die nur einmal COVID hatten. |
| 3 | Most people who repeatedly fall ill with COVID do not take care of their health. | 3 | Die meisten Menschen, die öfter an COVID erkranken, kümmern sich nicht um ihre eigene Gesundheit. |
| 4 | Most people who repeatedly fall ill with COVID do not follow expert medical advice. | 4 | Die meisten Menschen, die öfter an COVID erkranken, halten sich nicht an ärztlichen Rat. |
| 5 | Most people who repeatedly fall ill with COVID like to party or socialize often. | 5 | Die meisten Menschen, die öfter an COVID erkranken, feiern gerne und treffen sich oft mit anderen. |
| 6 | Most people who repeatedly fall ill with COVID are contaminated with germs. | 6 | Die meisten Menschen, die öfter an COVID erkranken, sind mit Keimen kontaminiert. |
| 7 | Most people who repeatedly fall ill with COVID are a burden to their families and society. | 7 | Die meisten Menschen, die öfter an COVID erkranken, sind eine Belastung für ihre Familien und die Gesellschaft. |
| 8 | Most people who repeatedly fall ill with COVID are socially irresponsible. | 8 | Die meisten Menschen, die öfter an COVID erkranken, sind sozial unverantwortlich. |
| 9 | Most people who repeatedly fall ill with COVID are a danger to other people. | 9 | Die meisten Menschen, die öfter an COVID erkranken, stellen eine Gefahr für andere Menschen dar. |

*Note.* Scale ranging from *strongly disagree* (1) to *strongly agree* (5); option *not applicable* available; original items from the COVID-PSS have been adapted from asking about “people infected with COVID” to asking about “people who repeatedly fall ill with COVID”; adaptations are underlined.

^a^ New, self-formulated items to assess behavioral aspects of stigmatization towards those infected with COVID-19 multiple times.

# **Table C** English and German items of the Long Covid Public Stigma Scale (LC-PSS)

|  | **English** |  | **German** |
| --- | --- | --- | --- |
| 1 | Persons with Long COVID are to blame for their problems. | 1 | Menschen mit Long COVID sind an ihren Problemen selbst schuld. |
| 2 | Long COVID usually results from a weak character. | 2 | Long COVID ist meistens die Folge eines schwachen Charakters. |
| 3 | Persons with Long COVID only have to pull themselves together in order to get well. | 3 | Menschen mit Long COVID müssen sich nur zusammenreißen, damit es ihnen wieder besser geht. |
| 4 | One of the main causes of Long COVID is lack of self-discipline. | 4 | Eine der Hauptursachen für Long COVID ist ein Mangel an Selbstdisziplin. |
| 5 | Long COVID is no real illness.^a^ | 5 | Long COVID ist keine echte Krankheit. |

*Note.* Scale ranging from 1 *strongly disagree* (1) to *strongly agree* (5); original items asking about “mental illness” have been adapted to ask about “Long COVID”.

^a^New, self-formulated item based on qualitative interviews with Long COVID patients who experienced that their illness wasn’t taken seriously.

# **Table D** English and German items of the Unvaccinated Public Stigma Scale (nonVac-PSS)

|  | **English** |  | **German** |
| --- | --- | --- | --- |
| 1 | I would be unhappy if someone who is not vaccinated against COVID married one of my close relatives. | 1 | Ich wäre nicht erfreut, wenn jemand, der/die nicht gegen COVID geimpft ist, eine*n meiner nahen Verwandten heiraten würde. |
| 2 | I would be afraid that someone who is not vaccinated against COVID infected me or my family with COVID-19. | 2 | Ich hätte Angst, dass jemand, der/die nicht gegen COVID geimpft ist, mich oder meine Familie mit COVID ansteckt. |
| 3 | I think someone who is not vaccinated against COVID is unintelligent. | 3 | Ich betrachte jemanden, der/die nicht gegen COVID geimpft ist, als nicht besonders intelligent. |
| 4 | I think someone who is not vaccinated against COVID is untrustworthy. | 4 | Ich halte jemanden, der/die nicht gegen COVID geimpft ist, für nicht vertrauenswürdig. |

*Note.* Scale ranging from *strongly disagree* (1) to *strongly agree* (5); option *not applicable* available; original items asking about “this person” based off of different vignettes was adapted to “someone who is not vaccinated against COVID”.

# **Table E** English and German items of the Covid-19 internalised stigma scale (COINS)

|  | Original Item |  | Translated item (German) |
| --- | --- | --- | --- |
| 1 | How much do you feel you are not as good a person as others because you have had COVID-19? | 1 | Wie sehr haben Sie das Gefühl, nicht so gut zu sein wie andere, weil Sie COVID hatten? |
| 2 | How much do you feel ashamed that you have had COVID-19? | 2 | Wie sehr schämen Sie sich dafür, dass Sie COVID hatten? |
| 3 | How much do you feel that it is your fault that you have had COVID-19? | 3 | Inwieweit glauben Sie, dass es Ihre Schuld ist, dass Sie COVID hatten? |
| 4 | How much do you feel embarrassed that you have had COVID -19? | 4 | Wie sehr ist es Ihnen peinlich, dass Sie COVID hatten? |
| 5 | How much have you avoided telling others that you have had COVID -19? | 5 | Wie sehr haben Sie es vermieden, anderen zu sagen, dass Sie COVID hatten? |
| 6 | How much have you stopped socialising with people because of their negative reactions to your having COVID-19? | 6 | In welchem Ausmaß haben Sie den Kontakt zu anderen Menschen abgebrochen, aufgrund deren negativen Reaktionen darauf, dass Sie COVID hatten? |
| 7 | How much do you feel uncomfortable to go anywhere outside of your house because you have had COVID-19? | 7 | Wie unwohl fühlen Sie sich, wenn Sie das Haus verlassen, weil Sie COVID hatten? |
| 8 | How much would you understand your family members if they avoided you because you have had COVID-19? | 8 | Wie sehr würden Sie Ihre Familienangehörigen verstehen, wenn diese Sie meiden würden, weil Sie COVID hatten? |
| 9 | How much would you understand friends if they avoided you because you have had COVID-19? | 9 | Wie sehr würden Sie Ihre Freund*innen verstehen, wenn diese Sie meiden würden, weil Sie COVID hatten? |
| 10 | How much would you understand your neighbours if they avoided you because you have had COVID-19? | 10 | Wie sehr würden Sie Ihre Nachbar*innen verstehen, wenn diese Sie meiden würden, weil Sie COVID hatten? |
| 11 | How much would you agree with employers if they would not employ you because you have had COVID-19? | 11 | Wie sehr würden Sie Arbeitgeber*innen zustimmen, wenn diese Sie nicht einstellen würden, weil Sie COVID hatten? |
| 12 | How much would you understand people if they excluded you from areas of public life (e.g. sporting centres, clubs, cultural events, voluntary groups) because you have had COVID-19? | 12 | Wie sehr würden Sie Menschen verstehen, wenn diese Sie aus Bereichen des öffentlichen Lebens (z. B. Sportstätten, Vereine, kulturelle Veranstaltungen, ehrenamtliche Gruppen) ausschließen würden, weil Sie COVID hatten? |
| 13 | How much would you understand doctor if he/she was not available to see you because you have had COVID-19? | 13 | Wie sehr würden Sie Ihren Arzt/ Ihre Ärztin verstehen, wenn er/ sie Sie nicht empfangen würde, weil Sie COVID hatten? |

*Note.* Scale ranging from *not at all* (1) to *a lot* (4); option *not applicable* was further available.

# **Table F** English and German items of the Unvaccinated Internalized Stigma Scale (nonVac-IS)

|  | **English** |  | **German** |
| --- | --- | --- | --- |
| 1 | Stereotypes about unvaccinated people apply to me. | 1 | Klischees über ungeimpfte Menschen treffen auf mich zu. |
| 2 | Negative stereotypes about unvaccinated people isolate me from others. | 2 | Negative Klischees über ungeimpfte Menschen isolieren mich von anderen. |
| 3 | I sometimes feel out of place because I am unvaccinated. | 3 | Ich fühle mich manchmal fehl am Platz, weil ich ungeimpft bin. |
| 4 | Being around people who are vaccinated makes me feel like I don't belong. | 4 | Unter Menschen zu sein, die geimpft sind, gibt mir das Gefühl nicht dazuzugehören. |
| 5 | Vaccinated people could not possibly understand me. | 5 | Geimpfte Menschen können mich unmöglich verstehen. |
| 6 | Nobody would be interested in getting close to me because I am unvaccinated. | 6 | Niemand wäre daran interessiert mir nahezukommen, da ich ungeimpft bin. |
| 7 | Being unvaccinated has negatively impacted my life.^a^ | 7 | Ungeimpft zu sein, hat mein Leben negativ beeinflusst. |
| 8 | In some social situations, I am embarrassed to be unvaccinated.^a^ | 8 | In manchen sozialen Situationen ist es mir peinlich, ungeimpft zu sein. |

*Note.* Scale ranging from *strongly reject* (1) to *strongly agree (4)*; original items asking about “mental illness” have been adapted to “unvaccinated”.

^a^New, self-formulated items oriented on further items of the original ISMI-29 (*Internalized Stigma of Mental Illness Scale, long form)*.

| **Table G** Response frequencies and percentages for the response options of the public stigma scales | | | | | | | | |
| --- | --- | --- | --- | --- | --- | --- | --- | --- |
| **Scale** | | ***n*** | **1 *n* (%)** | **2 *n* (%)** | **3 *n* (%)** | **4 *n* (%)** | **5 *n* (%)** | **-1 *n* (%)** |
| **COVID-PSS** | **Item 1** | 1960 | 709 (30,7) | 601 (30,7) | 471 (24,0) | 128 (6,5) | 51 (2,6) | n.a. |
|  | **Item 2** | 1960 | 475 (24,2) | 520 (26,5) | 668 (34,1) | 236 (12,0) | 61 (3,1) | n.a. |
|  | **Item 3** | 1960 | 550 (28,1) | 488 (24,9) | 648 (33,1) | 201 (10,3) | 73 (3,7) | n.a. |
|  | **Item 4** | 1959 | 586 (29,9) | 452 (23,1) | 554 (28,3) | 267 (13,6) | 100 (5,1) | n.a. |
|  | **Item 5** | 1960 | 611 (31,2) | 551 (28,1) | 542 (27,7) | 190 (9,7) | 66 (3,4) | n.a. |
|  | **Item 6** | 1960 | 730 (37,2) | 592 (30,2) | 408 (20,8) | 172 (8,8) | 58 (3,0) | n.a. |
|  | **Item 7** | 1960 | 758 (38,7) | 552 (28,2) | 443 (22,6) | 143 (7,3) | 64 (3,3) | n.a. |
|  | **Item 8** | 1959 | 429 (21,9) | 388 (19,8) | 644 (32,9) | 352 (18,0) | 146 (7,5) | n.a. |
|  | **Item 9** | 1960 | 611 (31,2) | 509 (26,0) | 468 (23,9) | 251 (12,8) | 121 (6,2) | n.a. |
|  | **Item 10** | 1960 | 769 (39,2) | 551 (28,1) | 410 (20,9) | 164 (8,4) | 66 (3,4) | n.a. |
|  | **Item 11** | 1960 | 403 (20,6) | 331 (16,9) | 505 (25,8) | 445 (22,7) | 276 (14,1) | n.a. |
| **MI-PSS** | **Item 1** | 1960 | 1328 (67,8) | 281 (14,3) | 156 (8,0) | 68 (3,5) | 40 (2,0) | 87 (4,4) |
|  | **Item 2** | 1960 | 1266 (64,6) | 305 (15,6) | 185 (9,4) | 87 (4,4) | 39 (2,0) | 78 (4,0) |
|  | **Item 3** | 1960 | 845 (43,1) | 412 (21,0) | 348 (17,8) | 166 (8,5) | 82 (4,2) | 107 (5,5) |
|  | **Item 4** | 1960 | 724 (36,9) | 372 (19,0) | 439 (22,4) | 192 (9,8) | 75 (3,8) | 158 (8,1) |
|  | **Item 5** | 1960 | 622 (31,7) | 341 (17,4) | 455 (23,2) | 242 (12,3) | 90 (4,6) | 210 (10,7) |
|  | **Item 6** | 1960 | 818 (41,7) | 310 (15,8) | 343 (17,5) | 173 (8,8) | 86 (4,4) | 230 (11,7) |
|  | **Item 7** | 1960 | 754 (38,5) | 385 (19,6) | 441 (22,5) | 212 (10,8) | 82 (4,2) | 86 (4,4) |
|  | **Item 8** | 1960 | 951 (48,5) | 415 (21,2) | 281 (14,3) | 146 (7,4) | 67 (3,4) | 100 (5,1) |
|  | **Item 9** | 1959 | 732 (37,4) | 361 (18,4) | 431 (22,0) | 261 (13,3) | 99 (5,1) | 75 (3,8) |
|  |  |  |  |  |  |  |  |  |

| **Scale** | | ***n*** | **1 *n* (%)** | **2 *n* (%)** | **3 *n* (%)** | **4 *n* (%)** | **5 *n* (%)** | **-1 *n* (%)** |
| --- | --- | --- | --- | --- | --- | --- | --- | --- |
| **LC-PSS** | **Item 1** | 1960 | 1378 (70,3) | 299 (15,3) | 202 (10,3) | 59 (3,0) | 22 (1,1) | n.a. |
|  | **Item 2** | 1960 | 1539 (78,5) | 227 (11,6) | 131 (6,7) | 44 (2,2) | 19 (1,0) | n.a. |
|  | **Item 3** | 1960 | 1271 (64,8) | 314 (16,0) | 233 (11,9) | 101 (5,2) | 41 (2,1) | n.a. |
|  | **Item 4** | 1960 | 1395 (71,2) | 276 (14,1) | 192 (9,8) | 68 (3,5) | 29 (1,5) | n.a. |
|  | **Item 5** | 1960 | 1287 (65,7) | 311 (15,9) | 183 (9,3) | 102 (5,2) | 77 (3,9) | n.a. |
| **nonVac-PSS** | **Item 1** | 1960 | 957 (48,8) | 270 (13,8) | 244 (12,4) | 156 (8,0) | 80 (4,1) | 253 (12,9) |
|  | **Item 2** | 1960 | 717 (36,6) | 261 (13,3) | 379 (19,3) | 302 (15,4) | 185 (9,4) | 116 (5,9) |
|  | **Item 3** | 1960 | 838 (42,8) | 245 (12,5) | 372 (19,0) | 254 (13,0) | 135 (6,9) | 116 (5,9) |
|  | **Item 4** | 1960 | 850 (43,4) | 361 (18,4) | 336 (17,1) | 187 (9,5) | 91 (4,6) | 135 (6,9) |

*Note.* Response options ranged from s*trongly disagree* (1) to *strongly agree* (5); for the MI-PSS and nonVac-PSS the response option *not applicable* (-1) was further available; COVID-PSS = Covid-19 Public Stigma Scale; MI-PSS = Multiple Infections Public Stigma Scale; LC-PSS = Long Covid Public Stigma Scale; nonVac-PSS = Unvaccinated Public Stigma Scale.

# **Table H** Response frequencies and percentages of the internalized stigma scales

| **Scale** | | ***n*** | **1 *n* (%)** | **2 *n* (%)** | **3 *n* (%)** | **4 *n* (%)** | **-1 *n* (%)** |
| --- | --- | --- | --- | --- | --- | --- | --- |
| **COINS** | **Item 1** | 1094 | 822 (75,1) | 75 (6,9) | 86 (7,9) | 40 (3,7) | 71 (6,5) |
|  | **Item 2** | 1094 | 925 (84,6) | 54 (4,9) | 43 (3,9) | 27 (2,5) | 45 (4,1) |
|  | **Item 3** | 1094 | 833 (76,1) | 130 (11,9) | 56 (5,1) | 29 (2,7) | 46 (4,2) |
|  | **Item 4** | 1094 | 923 (84,4) | 61 (5,6) | 40 (3,7) | 25 (2,3) | 45 (4,1) |
|  | **Item 5** | 1094 | 824 (75,3) | 123 (11,2) | 63 (5,8) | 35 (3,2) | 49 (4,5) |
|  | **Item 6** | 1094 | 742 (67,8) | 92 (8,4) | 74 (6,8) | 78 (7,1) | 108 (9,9) |
|  | **Item 7** | 1094 | 631 (57,7) | 133 (12,2) | 113 (10,3) | 87 (8,0) | 130 (11,9) |
|  | **Item 8** | 1094 | 604 (55,2) | 136 (12,4) | 107 (9,8) | 178 (16,3) | 69 (6,3) |
|  | **Item 9** | 1094 | 609 (55,7) | 131 (12,0) | 124 (11,3) | 168 (15,4) | 62 (5,7) |
|  | **Item 10** | 1094 | 610 (55,8) | 137 (12,5) | 122 (11,2) | 169 (15,4) | 56 (5,1) |
|  | **Item 11** | 1094 | 753 (68,8) | 79 (7,2) | 97 (8,9) | 74 (6,8) | 91 (8,3) |
|  | **Item 12** | 1094 | 737 (67,4) | 87 (8,0) | 110 (10,1) | 93 (8,5) | 67 (6,1) |
|  | **Item 13** | 1094 | 793 (72,5) | 76 (6,9) | 107 (9,8) | 72 (6,6) | 46 (4,2) |
| **nonVac-IS** | **Item 1** | 266 | 185 (69,5) | 52 (19,5) | 19 (7,1) | 10 (3,8) | n.a. |
|  | **Item 2** | 266 | 153 (57,5) | 59 (22,2) | 38 (14,3) | 16 (6,0) | n.a. |
|  | **Item 3** | 266 | 204 (76,7) | 39 (14,7) | 17 (6,4) | 6 (2,3) | n.a. |
|  | **Item 4** | 266 | 170 (63,9) | 62 (23,3) | 21 (7,9) | 13 (4,9) | n.a. |
|  | **Item 5** | 266 | 73 (27,4) | 72 (27,1) | 89 (33,5) | 32 (12,0) | n.a. |
|  | **Item 6** | 266 | 180 (67,7) | 63 (23,7) | 11 (4,1) | 12 (4,5) | n.a. |
|  | **Item 7** | 266 | 217 (81,6) | 37 (13,9) | 7 (2,6) | 5 (1,9) | n.a. |
|  | **Item 8** | 266 | 174 (65,4) | 49 (18,4) | 30 (11,3) | 13 (4,9) | n.a. |

*Note.* Response options for the COINS ranged from *not at all* (1) to *a lot* (4) and *not applicable* (-1) was further available as a response option; response options for the nonVac-IS ranged from *never* (1) to *often* (4); COINS = Covid-19 Internalised Stigma Scale; nonVac-IS = Unvaccinated Internalized Stigma Scale.

# **Table I** Goodness-of-fit indices for models with error covariances for the nonVac-IS

| **model** | ***n*** | **c*²* (df)** | **CFI** | **TLI** | **RMSEA** | **SRMR** | **AIC** | **BIC** |
| --- | --- | --- | --- | --- | --- | --- | --- | --- |
| 0 | 266 | 79.09 (20) | 0.90 | 0.86 | 0.10 | 0.06 | 4633.76 | 4691.09 |
| 1 | 266 | 65.38 (19) | 0.92 | 0.89 | 0.09 | 0.06 | 4622.05 | 4682.97 |
| 2 | 266 | 54.51 (18) | 0.94 | 0.91 | 0.08 | 0.05 | 4613.18 | 4677.68 |
| *Note.* Model 0 = baseline model with no modeled error covariance; model 1 = with error covariance between items 1 and 6; model 2 = with error covariances between items 1 and 6 and items 2 and 5; CFI = comparative fit index; TLI = Tucker-Lewis index; RMSEA = randomized standard error approximation; SRMR = standardized root-mean-square residual, AIC = Aikaike Information Criterion; BIC = Bayesian Information Criterion. | | | | | | | | |

# **Table J** Factor loadings of the nonVac-IS with modeled error covariances

| **Item** | **Standardized factor loadings** | |
| --- | --- | --- |
|  | **model 1** | **model 2** |
| 1 | 0.49 | 0.49 |
| 2 | 0.60 | 0.58 |
| 3 | 0.75 | 0.77 |
| 4 | 0.69 | 0.68 |
| 5 | 0.40 | 0.36 |
| 6 | 0.58 | 0.57 |
| 7 | 0.64 | 0.65 |
| 8 | 0.50 | 0.51 |

*Note.* Model 1 = with error covariance between items 1 and 6; model 2 = with error covariances between items 1 and 6 and items 2 and 5.

# **Table K** Response patterns for the option “not applicable”

|  | **MI-PSS**  (*n* = 1960) | **nonVac-PS**  (*n* = 1960) | **COINS**  (*n* = 1094) |
| --- | --- | --- | --- |
| Items answered  “not applicable” | ***n*** | ***n*** | ***n*** |
| 1 item | 152 | 145 | 132 |
| 2 items | 77 | 39 | 41 |
| 3 items | 51 | 23 | 16 |
| 4 items | 35 | 87 | 17 |
| 5 items | 22 |  | 9 |
| 6 items | 15 |  | 14 |
| 7 items | 7 |  | 14 |
| 8 items | 5 |  | 2 |
| 9 items | 27 |  | 2 |
| 10 items |  |  | 1 |
| 11 items |  |  | - |
| 12 items |  |  | 2 |
| 13 items |  |  | 20 |

*Note.* The pattern relates to the number of items on a scale answered with
“not applicable”. The MI-PSS contains nine, the nonVac-PS four, and the
COINS thirteen items; nonVac-IS = Unvaccinated Internalized Stigma Scale;
MI-PSS = Multiple Infections Public Stigma Scale; COINS = Covid-19
Internalised Stigma Scale.
